# Ethnic and gender comparison of rugae patterns among clinical dental trainees in Ibadan, Nigeria

**DOI:** 10.11604/pamj.2016.23.204.8584

**Published:** 2016-04-20

**Authors:** Bamidele Kolude, Adisa Akinyele, Ogunrinde Tunde Joshua, Lawal Ahmed

**Affiliations:** 1 Department of Oral Pathology, University of Ibadan and University College Hospital, Ibadan, Nigeria; 2 Department of Restorative Dentistry, University of Ibadan and University College Hospital, Ibadan, Nigeria

**Keywords:** Forensic, human, identification, palatal rugae, rugoscopy, Ibadan, Nigeria

## Abstract

**Introduction:**

This study was conducted to compare the rugae patterns between two major ethnic groups in Nigeria to establish any peculiarities. This will serve as basis for population identification especially in mass disasters involving individuals of different races or ethnicities.

**Methods:**

One hundred consenting participants, 50 of south-western Yoruba ethnicity and 50 of south-eastern Igbo ethnicity were recruited; impressions of the upper jaws were taken and cast with dental stone. Two blinded investigators then delineated and recorded the rugae pattern of individual casts. The rugae patterns for the two groups were then analysed using the SPSS version 16.

**Results:**

The Yoruba's had more of wavy and straight patterns while there were more of curve and circular among the Ibo's, however, there was no significant differences between the two groups in the mean incidence of the various rugae shapes of wavy, circular, curve and straight (p = 0.843, p = 0.711, p = 0.309 and p = 0.292 respectively). There were more secondary rugae in the Igbo than the Yoruba group and the differences in the mean incidences were significant.

**Conclusion:**

The study observed several rugae similarities and no significant differences in the primary rugae shapes of the Igbos and Yoruba ethnicities, however, there were significant differences in the sum of secondary and unclassified rugae between the two groups; therefore, rugoscopy may be useful in ethnic differentiation.

## Introduction

The Yoruba ethnic group are predominant in South-western Nigeria while the Igbo ethnic group are predominant in south-eastern Nigeria [1]. Yoruba ethnic group worldwide were estimated to be about 30 million in the year 2010, while there were approximately 24 million of Igbo ethnicity [2]. Global distribution of these two ethnic groups is as a result of slave trade, educational and business determination. Palatal rugae consist of a fibrous connective tissue core embedded deeply between the submucosal fatty tissue and the stratum reticulum of the palate [3]. This core represents the foundation over which the substance of palatal rugae builds to become a fold-like protuberance in the palate [4]. A previous study [5] in similar population had reported that none of the hundred individuals studied had similar rugae in terms of the shape and length, and this supports the uniqueness of palatal rugae pattern as a tool for human identification. The varying shapes of palatal rugae can be attributed to the fact that rugae develop as localized regions of epithelial proliferation and thickening. Fibroblasts and collagen fibers then accumulate in the connective tissue beneath the thickened epithelium and assume distinct orientation [6]. Although individuals show different characteristics, the question of similarities in tribes arise, this also will be determined by the use of morphology and length of the rugae. Morphology varies from wavy, curved, circular, straight to unified. Curved: a crescent shape that is curved gently. Wavy: If there is a slight curve at the origin or termination of a curved rugae. Straight: They run directly from their origin to termination. Circular: Rugae that form a definite continuous ring. Unification occurs when two rugae join at their origin or termination. Diverging: If two rugae had the same origin from the midline but immediately branched. Converging: Rugae with different origins from midline, but which joined on their lateral portions [6]. The palatal rugae pattern is unique to humans and may be specific to ethnic groups and/or gender hence the suggestion for its use in population and gender identification in forensic dentistry [7]. These rugae patterns are well protected by the lips, the buccal pad of fat and teeth. They are said to be stable throughout life following completion of growth and they can be used effectively in post mortem identification provided an ante-mortem record exists [8]. Human identification is required for certification of death and for personal, social, legal and humanitarian reasons [9]. This becomes particularly important in mass disasters involving individuals of different races or ethnicities [9]. In the background of few studies on the forensic role of palatal rugae in West Africa, we aim to compare the rugae patterns between two major ethnic groups contrasted with gender in Ibadan, Nigeria to establish any peculiarities.

## Methods

One hundred consenting participants, 50 of south-western Yoruba ethnicity and 50 of South-Eastern Igbo ethnicity, were recruited into the study. This consisted of 50 male and 50 female clinical dental trainees aged between 20-30 years. Informed verbal consent was obtained from all participants. Subjects with prominent palatal tori and those using dentures were excluded from the study. All participants had impressions of the upper jaw taken with alginate impression material (Tub-Henry schein-Regular Set) loaded into upper full arch metal stock trays. The impressions were taken by the same operator to correct for internal errors of technique. All impressions were cast immediately with dental stone (Dentsply-Trubyte) and numbered sequentially. Ethical approval (AD13/479/352) was obtained from Oyo state research ethical review committee. Two blinded investigators then delineated the rugae pattern of individual casts with a sharp lead 2B pencil. Rugae less than 5mm in length were described as secondary, while those up to 5mm or more in length were described as primary rugae. The patterns were sub-classified into wavy, curved, circular, straight, divergent and convergent shapes according to the modified Thomas and Kotze criteria [10]. All analyses were done using the SPSS version 16.

## Results

The sample studied comprised 100 dental stone models representing 50 Yoruba (group A) and 50 Igbo (group B) participants. There were 25 males and 25 females in each group. The overall mean age was 22.7 ± 2.5 years, the mean ages of males and females were 23.1 ± 2.6 years and 22.2 ± 2.4 years respectively; there was no significant difference in the mean ages of male and female participants (p = 0.088). The mean ages of group A and B participants were 22.5 ± 2.4 and 22.8 ± 2.6 years respectively, there was also no significant difference in the mean ages of group A and group B participants (p = 0.66). Wavy, circular, curve and straight were the predominant shapes in the present study with incidence and mean of 29.6% (3.5 ± 1.5); 21.4% (2.9 ± 1.8); 20.7% (2.7 ± 1.3) and 19.8% (2.6 ± 1.2) respectively while divergent, convergent and unclassified rugae types were rare with incidence and mean of 3.3% (1.3 ± 0.6), 3.0% (1.1 ± 0.4) and 2.2% (1.1 ± 0.4) respectively. Overall there were significant differences between the incidence of wavy and curve; wavy and circular and wavy and straight rugae patterns (p = 0.000; p = 0.012 and p = 0.000 respectively), but there was no significant difference between the overall incidence of curve and circular; curve and straight, and between circular and straight patterns (p =0.169; p = 0.071 and p = 0.659 respectively). Furthermore, there were no significant differences between the two groups in the mean incidence of the various rugae shapes of wavy, circular, curve and straight (p = 0.843, p = 0.711, p = 0.309 and p = 0.292 respectively) Table 1. There were also no significant differences between gender in the mean incidence of the various shapes of wavy, circular, curve and straight (p = 0.27, p = 0.452, p = 1.0 and p = 0.572 respectively) Table 2. Primary pattern was predominant and there was a significant difference in the overall mean incidence of primary rugae pattern ( = 8.71) when compared with the secondary pattern ( = 3.16) (t = 18.9; p =0.000). Group A had more of wavy and straight patterns while there were more of curve and circular in group B. Females had more of wavy, circular and curve shapes while males had more of straight shape. However, there were no significant differences between the two groups and between both sexes in the incidence of primary pattern (p = 0.312 and p = 0.193 respectively) (Table 1 and Table 2). In addition, there was no significant difference between both sexes in the incidence of secondary rugae (p = 0.368). However, there was a significant difference in the mean incidence of secondary rugae that was more in the Igbo than the Yoruba ethnicity (p = 0.00) (Table 1). In general diverging rugae patterns superseded converging and were more in females and group A participants, however the differences were insignificant. Furthermore, the convergent and the divergent unification patterns showed no significant differences between the two groups (p = 0.348 and p = 0.931 respectively) and between both genders (p = 0.348 and p = 0.622). There was also no significant difference between both genders in the mean incidence of unclassified rugae (p = 0.361). However, there was a significant difference in the mean incidence of unclassified rugae between group A and B (p = 0.041).

**Table 1:** Ethnic comparison of rugae types

	Total incidence (N)	Total rugae	Group A incidence (n)	Group A rugae sum	Group B incidence (n)	Group B rugae sum	t-test	p- value	Remark
Wavy	113	406	58	204	55	202	0.74	0.46	NS
Curve	102	263	51	122	51	141	-1.48	0.14	NS
Straight	101	250	49	135	52	115	1.10	0.27	NS
Circular	93	265	48	126	45	139	-.27	0.79	NS
Primary	120	1018	60	499	60	519	-.90	0.37	NS
Secondary	94	284	47	94	47	190	5.23	0.00	S
Convergent	39	44	18	22	21	22	-1.27	0.22	NS
Divergent	35	47	18	24	17	23	0.67	0.51	NS
Unclassified	25	29	15	10	10	19	-2.26	0.04	S

NS = Not significant; S = Significant

**Table 2:** Gender comparison of rugae types

	Total incidence(N)	Total rugae	Male incidence(n)	Male sum of rugae	Female incidence (n)	Female sum of rugae	t-test	p- value	Remarks
Wavy	113	406	53	201	60	205	0.57	0.57	NS
Curve	102	263	45	118	57	145	1.13	0.26	NS
Straight	101	250	52	132	49	118	1.49	0.14	NS
Circular	93	265	42	129	51	136	1.05	0.30	NS
Primary	120	1018	55	483	65	535	1.24	0.22	NS
Secondary	94	284	44	142	50	142	0.31	0.76	NS
Convergent	39	44	19	20	20	24	-1.13	0.27	NS
Divergent	35	47	11	15	24	32	0.14	0.89	NS
Unclassified	25	29	8	10	17	19	0.82	0.42	NS

NS = Not Significant

## Discussion

Palatal rugae have been studied for various reasons, the most important one being for personal identification in the field of forensic odontology. Several investigators [11–14] have recognized palatal rugae pattern to be individually distinct. It has also been established that rugae maintains a constant shape throughout life [14] and may be specific to racial groups thus facilitating population identification [2, 15]. The most common rugae shape in the present study was the wavy pattern followed by the circular, curve and straight. Previous palatal rugae studies among ethnic groups have indicated that the wavy pattern is the most frequent shape [15–18]. Contrary to this finding, Gondivkar et al [19] in a study among western Indians, observed the curve shape as the most common followed by wavy rugae types, while Eboh [20] in a Nigerian study observed straight shape as the most common followed by wavy. Although there are recognizable racial variations, the pattern of rugae is obviously very useful for personal and racial identification because of the varying shape proportion and sequences. The circular rugae shape has also shown wide variation, with some Indian authors [15] recording a zero incidence while other Indian authors like Paliwal et al [21] and Bharath et al [22] recorded a low presence of circular patterns in the primary rugae region. There was a low incidence of circular rugae shape in the study of Eboh [20] among south-eastern Nigerians but Hauser et al [23], reported a high incidence of circular shapes among the Swazi tribe of South Africa when compared with Greeks. In the present study, there was a relatively high incidence of circular rugae shape that ranked next in occurrence to the wavy pattern; this supports the observation of Hauser et al [23] among black South Africans. However, Thomas and Kotze [24] in a study of two genetically similar populations stated, “the race of individuals cannot be determined with certainty using rugae”. Shetty et al [25] also found no significant difference in the total sum of rugae between the Tibet and Mysore ethnic groups of India. Palatal rugoscopy discrimination between populations is factored on wide genetic and geographic separation [15] and the rugae classification method adopted for the study [17, 20]. The role of genetics in rugae pattern is typified on one hand by the extremely close palatal rugae features in identical twins while on the other hand widely separated and racially different population often present many dissimilar rugae features [12, 16, 20]. Some authors [16, 18, 24, 26] stated that palatal rugoscopy was not useful for gender discrimination while others [19, 23] reported a significant role for palatal rugoscopy in gender discrimination. Yamazaki [27] in a Japanese study and Reuer [28] in an Australian study suggested sexual dimorphism in the biometric features of palatal rugae. Dohkhe et al [29] in a Japanese study of the palate observed that it was variation in the secondary rugae that led to significant differences in rugae shapes between the genders. Although in the present study there was no significant difference in the rugae incidence between genders either in the primary or secondary palate, there was a significant difference in the occurrence of secondary and unclassified rugae between the Igbo and Yoruba ethnicity. Thomas et al [24] suggested the use of discrete variables of shapes rather than measurement of rugae dimension which is a continuous variable that has minimal discriminatory power. In the present study, the primary rugae failed to establish any difference between the two genders and population groups but a significant difference was observed in the secondary rugae between the two population groups although this was not the case between genders. Generally, populations that are genetically and geographically close, as in the present study, where neighbouring populations with centuries of social interaction that include inter-marriages tend to feature several similar rugae features. However the less prominent secondary and unclassified rugae such as breaks and papillation featured significant differences that were useful in distinguishing the Igbo from Yoruba ethnicity. A major limitation in the use of rugoscopy in ethnic differentiation is the wide variation and the lack of a universal standard criterion in classification of palatal rugae shape. This might have accounted for some differences in the present study that utilised modified Thomas and Kotze [24] classification and that of Eboh [20] that utilised Trobo's classification since the two studies were conducted in the same geographic region.

## Conclusion

The present study observed several rugae similarities and no significant differences in the primary rugae shapes of the Igbos and Yoruba ethnicities that are domiciled in south eastern and south western Nigeria respectively. However, there were significant differences in the sum of secondary and unclassified rugae between the two ethnicities. There was no significant difference in the primary, secondary and unclassified rugae patterns between the male and female genders; therefore, rugoscopy was useful in ethnic differentiation but not in gender separation.

### What is known about this topic

- The palatal rugae has been studied and found to be individually distinct;
- The palatal rugae has been suggested to be specific for racial groups.

### What this study adds

- Palatal rugae was studied among Yoruba an Ibo ethnic group for the first time in Nigeria and we found the wavy pattern to be most consistent in these two ethnic groups;
- We found no significant differences in primary rugae patterns of the two groups;
- There were significant differences in secondary and unclassified rugae patterns between the two ethnic groups.
